# Families tackling adolescent anorexia nervosa: family wellbeing in family-based treatment or other interventions. A scoping review

**DOI:** 10.1007/s40519-024-01641-z

**Published:** 2024-03-20

**Authors:** Signe Holm Pedersen, Dorthe Andersen Waage, Nadia Micali, Mette Bentz

**Affiliations:** 1 Child and Adolescent Mental Health Center, Copenhagen University Hospital-Mental Health Services CPH, Copenhagen, Denmark; 2 Center for Eating and Feeding Disorders Research, Mental Health Center Ballerup, Copenhagen University Hospital - Mental Health Services CPH, Denmark, Ballerup, Denmark

**Keywords:** Family-based treatment, Anorexia nervosa, Family function, Family relations, Attachment, Carer burden

## Abstract

**Purpose:**

Family-based treatment (FBT) has contributed significantly to the treatment of anorexia nervosa (AN) in young people (YP). However, parents are concerned that FBT and the active role of parents in the task of refeeding may have a negative impact on family relations. The aim of the review is to assess whether families engaged in FBT for AN are more or less impacted in their family wellbeing and caregiver burden, compared to families with a YP diagnosed with AN, who are not undergoing treatment with FBT.

**Method:**

Computerized searches across six databases complemented by a manual search resulted in 30 papers being included in the scoping review.

**Results:**

The review identified 19 longitudinal studies on change in family wellbeing in families in FBT-like treatments, and 11 longitudinal studies on change in family wellbeing in treatment where parents are not in charge of refeeding. Only three randomized controlled studies directly compare FBT to treatment without parent-led refeeding.

**Conclusion:**

The available research suggests no difference between intervention types regarding impact on family wellbeing. Approximately half of the studies find improvements in family wellbeing in both treatment with and without parent-led refeeding, while the same proportion find neither improvement nor deterioration. As parents play a pivotal role in FBT, there is a need for good quality studies to elucidate the impact of FBT on family wellbeing.

**Level of evidence** *Level V:* Opinions of authorities, based on descriptive studies, narrative reviews, clinical experience, or reports of expert committees.

**Supplementary Information:**

The online version contains supplementary material available at 10.1007/s40519-024-01641-z.

## Introduction

Anorexia nervosa (AN) is a serious disease, typically debuting in the early teenage years [1]. Historically AN has been viewed as caused by maladaptive family process as reflected in Minuchin’s concepts of the psychosomatic family [2]. During the 1990s, perspective of parents gradually changed and The Maudsley hospital in London developed a family therapy (Maudsley FT), where parents were perceived as the most important resource in relation to fighting AN in young people (YP). The rationale for the treatment was to circumvent the affected YP’s fluctuating treatment motivation by supporting parents in taking responsibility for YP refeeding. The treatment was later manualized by Lock and Le Grange as Family Based treatment (FBT) [3], and exists in both the strict manualized version and in the Maudsley FT version, which stresses more engagement of YP. For the sake of readability, this article does not distinguish between these two versions. Furthermore, other variations such as multifamily therapy (MFT) [4] and parent-only therapy [5] have been developed. All versions and variations have parents in charge of refeeding as the central component.

Today FBT is the recommended first line of treatment for Anorexia Nervosa (AN) in youths worldwide [6–8]. FBT is rooted in systemic and behavioral therapeutic approaches and has parent empowerment as the main therapeutic focus. Young people are treated in their own home, parents are instructed to take responsibility for YP eating, prevent disturbed behaviors and secure stable weight gain [3].

FBT has contributed significantly to the treatment of AN, a disorder that otherwise has poor prognosis and high mortality [1]. However, some parents report that FBT and the task of refeeding in the face of strong emotions have detrimental effects on the relationship with their child and on family functioning [9]. Indeed, a growing body of studies highlights the enormous carer burden of parents of a YP with AN as well as a general reduced family function in affected families, regardless of treatment approach [10]. However, it is unknown whether the effects on families are larger or more pronounced in families who undertake FBT than in families who undertake other treatments or no treatment at all for AN in YP. For example, having the additional strain of taking an active role in treatment might increase the burden on parents or families. Alternatively, having an active role in treatment and experiencing overcoming a serious disorder together as a family might have the potential to strengthen the bond between child and parents.

Although FBT helps more YP recover from AN than other available treatments, it is important to understand potential iatrogenic effects of FBT on family wellbeing for several reasons. Firstly, family function is pivotal for children’s cognitive and emotional development [11]. Secondly, research has established an association between affected family wellbeing and poorer outcome of treatment of AN [12]. A step towards counteracting potential iatrogenic effects on the family is to clarify to what degree potential effects are due to treatment components specific to FBT or to factors related to the burden of having a YP with AN irrespective of treatment approach.

The term “family wellbeing” is a broad, common sense or lay term implying a view of the family as a social unit or system where the relationships and all other aspects of the members´ functioning impact the lives and health of its members [13]. We have operationalized “family wellbeing” as encompassing the common-sense concepts of family function, family relations, carer burden and parental stress, as well as the theoretical construct of attachment. Although not a common-sense term, we included attachment as a specifier of the relationship between YP and parents because parents and clinicians have voiced the concern that FBT may harm the essential attachment between YP and parents [14].

Before embarking on a systematic review compiling measurements of each of these aspects of family wellbeing separately, a scoping review may provide an overview of what is known across many aspects of family wellbeing. We limit the scope of effect to the YP with AN and the parents, excluding studies focusing on siblings. We acknowledge that siblings are an integral part of a family, and their wellbeing is equally important, but the influence of AN on siblings form an entire field of study in itself.

To the best of our knowledge, no review has mapped effects on family wellbeing and carer burden in families with a YP with AN in treatment with FBT in relation to the effects on these phenomena in families with a YP with AN *not* in FBT.

The objective of the present scoping review is to provide an overview of the current state of knowledge on whether families in FBT (or similar treatment, where parents are systematically advised to take responsibility for YP´s refeeding) are more or less affected in their family wellbeing over time than families with a young one with AN NOT in treatment with FBT.

## Methods

The review is organized as a PICO study, comparing an intervention arm with a comparison arm. Population: families with a YP diagnosed with AN or atypical AN according to either ICD 9, 10, 11 or DSM III, IV or 5. Intervention: family-based treatment or similar therapy with parent-led refeeding. Comparison: families in other types of treatment or not in treatment at all. Outcome: family function, family relations, carer burden, parental stress, and attachment.

Inclusion criteria: Longitudinal quantitative studies published in peer reviewed journals written in English, measuring any aspects of family function, family relations, carer burden, parental stress and/or attachment between a YP with AN and his/her parents, with a minimum of two measurement time points. As the world Health Organization (WHO) define youth as individuals aged 10–19 years, we define YP as less than 20 years of age [15]. Exclusion criteria: Qualitative studies and single case studies.

Searches were performed in PubMed, Embase, PsycInfo, Web of Science, Scopus and Cinahl in March 2023 and imported to CADIMA [16]. Reference lists of relevant papers and identified reviews were searched for additional relevant papers. When necessary, study investigators were contacted.

All papers were evaluated based on title and abstract, and potentially relevant papers were subjected to a full text screening. Disagreements on study selection were resolved by discussion until consensus was reached. See Table S1 and S2 in supplementary materials for the data extracted. Studies were not subjected to a critical appraisal of validity and risk of bias, as the review objective was to provide an overview of the current state of knowledge and not a synthesized result. The protocol was drafted using the Preferred Reporting Items for Systematic Reviews and Meta-analysis Protocols—extensions for scoping reviews [17].

Studies included in the review were divided into three conditions: (a) longitudinal studies where YP had not been in treatment at all (b) longitudinal studies where families had been in FBT or similar treatment with parent-led refeeding. This category includes different types of treatments such as classic FBT, Maudsley FT, separated family therapy, parent-focused therapy, multifamily therapy and other treatment types, having in common that parents are systematically instructed to take responsibility for YP refeeding (c) longitudinal studies where families had been in treatment with *no* systematic parent-led refeeding. This category includes a range of different therapies such as generic systemic family therapy, individual cognitive behavioral therapy, etc., all having in common, that parents are *not* systematically instructed to take responsibility for YP refeeding. These studies are hereafter referred to as “Other treatments”).

## Results

**Fig. 1:**
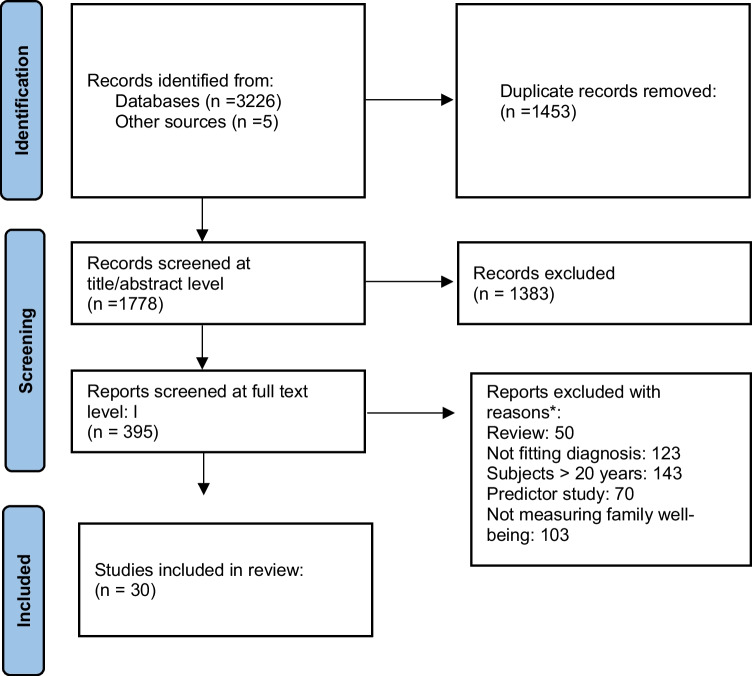
Prisma flow diagram. *Some records excluded due to several reasons

The initial search of the databases resulted in 3226 studies. Five additional studies were identified through other sources, resulting in 3231 studies being exported to the web tool CADIMA [16]. First 1453 duplicates were removed. Then 1383 studies were excluded on title/abstract level leaving 395 studies to be screened on full text level. The screening resulted in 30 studies being included in the review. See PRISMA flow-diagram in Fig. 1.

During the data extraction process, it became clear that a number of studies have operationalized family wellbeing with the concept of Expressed Emotion (EE). EE is a measure of a relative’s attitudes and behaviors toward an ill family member and consists of five components: critical comments (CC), hostility (HOS), emotional overinvolvement (EOI), warmth, and positive remarks (POS). High EE are demonstrated to predict dropout and poorer outcome across a number of disorders [18]. Studies using EE as a measure of family wellbeing will be reported under the heading family relations.

Some studies measure more than one aspect of family wellbeing. For the sake of overview, each aspect will be reported separately under the relevant heading.

Further, when mapping out the literature, we discovered that carer burden and parental stress were treated somewhat interchangeably, the terms henceforth are reported combined.

### A: Studies with no treatment

We identified no longitudinal studies on family wellbeing in families with a YP with AN not in treatment.

### B: FBT or similar treatment with parent-led refeeding

Nineteen studies involved FBT or similar treatment (such as classic FBT, Maudsley FT, separated family therapy, parent-focused therapy, multifamily therapy, etc.), all treatments where parents were systematically advised to take responsibility for YP refeeding. Of these, seven reported findings from randomized controlled trials (RCT), two from controlled longitudinal studies, and ten from longitudinal studies without a comparison-group (see Table S1, supplementary materials). Only three RCTs compared treatment with parental-led refeeding with treatment where parents were not systematically advised to take charge of refeeding. The remaining four RCTs compared different variations of treatment with parents in charge of refeeding. 16 studies examined change in family wellbeing *in pure* outpatient samples; however, a large proportion of participants in these studies had a prior hospitalization. Three studies consisted of a mixture of in, day- and out-patients. Sample sizes varied from 18 to 210 participants, with the majority of studies being rather small. Most studies assess family wellbeing at baseline and at EOT, however some studies focus on change in the first months of treatment of during treatment, while others also include data from follow-up.

#### Family functioning

The loosely defined concept of family function encompasses family cohesion, adaptability, levels of conflict, and organization and quality of communication [19].

The review identified seven studies (reported in eight papers) examining change in family functioning after treatment for AN with parent-led refeeding. Family functioning was measured with the Family Assessment Device (FAD) [20] or the Family Adaptability and Cohesion Evaluation Scales (FACES III) [21]. Overall, two uncontrolled studies found overall improvement in family functioning [4, 22], two RCTs [19, 23] and one uncontrolled study [24, 25] found no change in family functioning. None of the studies found an overall deterioration. One RCT found both improvement and deterioration on subscale levels [26]. Finally, one controlled study [24, 27] found both improvement, unchanged level and deterioration depending on respondents.

Looking at findings in more detail, one RCT [19] compares FBT and adolescent-focused therapy (AFT) (where the YP was encouraged to take responsibility for own recovery) and found no change in family functioning in any of the treatments. Two RCTs [23, 26], with one being a pilot for the other, compared Conjoint Family Therapy (CFT) and Separated Family Therapy (SFT) with parents in charge of refeeding in both conditions. The pilot and the later study differed in duration of treatment only but found differing results. Hence, while the pilot RCT found no change in family functioning in any of the treatments, in the later RCT [26] families rated themselves as less enmeshed after both treatment conditions. However, while YP in CFT rated their families as more adaptable or flexible, YP in SFT rated their families as less adaptable and more rigid over time.

One controlled study, reported in two papers, compared families with a YP with AN in outpatient FBT, with families with a healthy YP (HC) and found no change in family functioning for either YP with AN or HC. However, mothers of youths with AN reported improvement of family functioning, whereas fathers in both groups reported a deterioration [24, 27].

Of three uncontrolled studies [4, 22, 25] one study on FBT for transition age youth found perceived family functioning to improve after treatment [22]. Similarly, perceived family functioning improved from baseline to end of treatment (EOT) and remained improved at follow-up in a study on multi-family therapy (MFT) [4]. On the contrary, a study on modified FBT found no effect on perceived family functioning [25].

#### Family relations including EE

Ten studies, of which four are RCTs, reported change in family relations. Of these, two measured family relations conceptualized as family climate and communication, and eight measured family relations conceptualized as EE. Overall, the studies on family climate and communication found perceived family climate and communication, as well as observed family communication to improve, while the studies on EE found no change.

Family climate and communication were measured with the Parent Adolescent Relationship Questionnaire (PARQ) [28] (YP and parent self-rating), and by a family-interview (observer rating).

EE was measured with the Family Questionnaire (FQ) [29] (YP and parent self-rating), the Standardized Clinical Family Interview (SFCI) [30] (observer-rating), or the Five Minute Speech Sample (FMSS) [31] (observer-rating).

Looking at findings regarding climate and communication in more detail, one RCT compared behavioral family systems therapy (BFST) or ego-oriented individual therapy (EOIT). In BFST parents were asked to take responsibility for YP’s refeeding, whereas in EOIT parents were instructed to *refrain* from involvement in YP’s eating [32, 33]. The study found significant reductions in both perceived and observed negative communication and parent-adolescent conflict after both treatments. Similarly, an uncontrolled study on Maudsley inspired family therapy found observed family climate to improve after therapy [34]. One additional study [12] measured family climate before and after FBT using a self-report questionnaire. However, general change was not reported, nor available via contact with the author.

Looking at findings regarding EE in more detail, none of three RCTS and five uncontrolled studies found an overall change in EE. However, some of the studies pointed to respondent-dependent change in general EE or EE subcategories. Hence, a larger RCT compared conjoint FBT with parent-focused FBT (PFT) and found PFT to be associated with a decrease (improvement) in observed maternal CC, whereas an increase (worsening) in maternal CC were more likely in FBT than in PFT [35]. In the same line, a pilot-RCT comparing conjoint family therapy (CFT) with separated family therapy (SFT), with parents in charge of refeeding in both conditions found CC to increase in CFT and decrease in SFT [23]. However, authors suggested this might be due to a few extreme scores given the small number of participants. In the subsequent RCT, significant reductions in CC were found in both treatment conditions [26].

Five uncontrolled studies yielded mixed results. A large study [36] pooling together families with a YP in either multidisciplinary day- or inpatient treatment found an improvement (i.e., reduction) in both perceived emotional overinvolvement (EOI) and in perceived critical comments (CC) from baseline to EOT. However, CC increased after EOT, and at 2 years FU it nearly reached baseline level. A smaller study [37] also found perceived EOI, but not CC to decrease significantly after 6 months of FBT. One study [38] rated observed numbers of neutral in-session statements from parents to child, which may be viewed as an aspect of low EE. The study reported change between sessions 1 and 4 of FBT for early responders and not-early responders separately and found neutral comments to increase in both groups [34]. On the contrary, a study on FBT for transition age youth [22] found perceived EE *not* to improve after FBT. In line with this, a small study [39] found that in the few families with high observed EE at start, family therapy did not seem to help the families in lowering the level of EE. One study measured EE before and after FBT in a partial hospitalization program, however change in EE was not reported, nor available via contact with the author [40].

#### Carer burden and parental distress

Four studies explored concepts related to the burden or distress parents may experience during FBT treatment. This ranges from the individual parent’s belief about their capacity to execute behavior, conceptualized as self-efficacy (SE), to how burden is experienced and may be targeted during treatment. Burden has been measured as both a distinctive concept with the Burden Assessment Scale [41] and the Eating Disorder Impact Scale (EDSIS) [42] and through measurements of depressive, anxiety and stress symptoms measured with the Beck Depressive Inventory [43]. SE has been self-reported by parents using the Parent Versus Eating Disorder scale [44] and the General Self-Efficacy Scale [45]. Overall, two studies (one controlled and one uncontrolled) found SE to increase during FBT, and one of these also found SE to be negatively linked to carer burden. One controlled study found depressive symptoms in parents to decrease during FBT and finally one controlled study found accommodation to decrease during treatment.

Looking at results in more detail, one randomized parallel trial and an uncontrolled study explored caregiver SE in families treated with FBT. One randomized parallel trial found perceived SE to increase in parents in FBT but not in Systemic Family Therapy (SyFT) [46]. The authors suggest this may be understood as a result of the different therapeutical targets, where FBT is designed to foster parental empowerment while SyFT mainly targets miscommunication and family processes. The second study adds to these findings, and found caregiver SE to increase and carer burden to decrease after FBT delivered as telemedicine [44].

One study investigated associations between accommodation behavior and carer distress [47]. The treatment delivered was not solely based on FBT, but a family-centered, symptom-oriented, partial hospitalization program with similar responsibility given to parents in regard of the child´s food intake. Although this study found perceived levels of accommodation to decrease during treatment, changes in distress were not reported [47].

One uncontrolled study explored depressive symptoms in parents, where the treatment was described as family-oriented and based upon weight restoration, however, not strictly based on the FBT manual [36]. Up to half of the mothers in the study had high levels of perceived depressive symptoms at admission, corresponding to a mild depression. These levels had decreased by discharge and at follow-up 2.5 years later but stayed elevated compared to community norms.

#### Attachment

One study, reported in two papers, compared change in YP’s perceived attachment to parents after FBT with change in perceived attachment in healthy YP (HC). The study found perceived attachment to decline from baseline to EOT and remain unchanged at 14 months follow-up (FU) in both groups [24, 27].

### Family wellbeing in FBT-like treatment: summing up

*Overall*, results from the 19 studies on change in family wellbeing in families in FBT-like treatments point to family wellbeing either improving or remaining unchanged but not deteriorating. Looking at the different phenomena of family wellbeing separately, family function, family relation/climate, carer burden and parental distress seem more prone to improve with approximately half of these studies finding an improvement in family wellbeing, while the other half find an unchanged level. On the contrary, levels of general EE remain unchanged in all studies. Finally, attachment decrease in both YP with AN and in HC, indicating this to be a normal tendency during adolescence.

Three RCT studies directly addressed the review question by comparing FBT-like treatment to non-FBT-like treatment. They found unchanged level of family function [19], and a reduction in negative communication and conflict [32, 33] regardless of treatment type. Parental SE increased in FBT-like treatment, but not in non-FBT-like treatment [46]. These studies did not address EE, nor attachment.

### C: Other treatments

Ten studies (11 papers) assessed change in family wellbeing after treatment *without* parent-led refeeding. This category pools together a range of therapies such as individual CBT, generic systemic family therapy, etc., all having in common that parents are not systematically asked to take over YP refeeding. Of the studies in this category 5 report findings from RCTs, 2 from controlled longitudinal studies, and 4 from longitudinal studies without a comparison-group. Three studies consisted of inpatients, four of outpatients, one of a mixture of in- and out-patients and two studies did not specify patient status. Sample size varied from 11 to 233 participants (see Table S2, supplementary materials). Most studies assess family wellbeing at baseline and at EOT, however a few studies also report changes during therapy as well as at follow-up.

#### Family functioning

Four studies examined change in family function after treatment without parent-led refeeding. Perceived family function was measured with FAD [20] or FACES III [21], completed by YP and parents or YP and mothers. One study used observer-ratings of family function from video-recorded family interactions. Overall, studies found a general improvement in family functioning after some, but not all treatments. Furthermore, two studies found a mixed pattern of respondent-dependent change.

Looking at findings in more detail, two RCTs found improvement in family functioning at FU for all assessed family members after three treatment types (individual CBT, behavioral family therapy, and systemic family therapy) but not after treatment as usual (TAU) [48, 49]. Family function also improved at FU in former inpatients randomized to systemic family therapy (not focused on ED symptoms) as an addition to TAU, but not after TAU.

Two studies (one controlled and one uncontrolled) found mixed informant-dependent results without a clear pattern. The largest study compared perceived family function in inpatients and their parents allocated to either TAU plus multifamily group with YP *present* or multifamily group with *parents only* [50]. The overall treatment aim of the multifamily groups was to establish autonomy and self-determination of young people and not parent-led refeeding. Only fathers reported a general improvement in family functioning across both treatments. Problem-solving improved according to YP but deteriorated according to mothers—authors suggest this might be due to treatment focusing on autonomy of the YP. Another uncontrolled study consisting only of YP and mothers, found YP perceived family functioning to deteriorate after 1 year of an unspecified treatment, followed by an improvement at two years FU. Mothers reported no changes in family functioning. Results are reported in two papers [51, 52].

#### Family relations including EE

One controlled and two uncontrolled studies consisting of inpatients only examined change in family relations, including EE after treatment where parents were not in charge of refeeding. Family climate and communication were measured with observer ratings of recorded therapy sessions [53]. EE was measured with Level of Expressed Emotion Scale (LEE) [54] or FQ.

These studies found (a) an improved communication style with aggression being more open instead of covert [53] and (b) no change in EE [29, 55].

Looking at findings regarding family climate and communication in more detail, one uncontrolled study examined change in communication style after systemic family therapy focusing on dysfunctional behavioral patterns in the family. The study found a significant change during therapy from covert aggression to overt communication of aggression, reflecting a movement to a more open communication style [53].

Looking at findings regarding EE in more detail, a controlled study compared changes in EE after TAU to changes after TAU plus a supplementary parent-support intervention. No significant changes in EE after any treatment or support intervention were identified [29]. Correspondingly, in a multicentre trial YP rated perceived EE from parents at admission to inpatient stay and at discharge and reported no changes in level of perceived EE [55].

#### Carer burden and parental distress

Numerous studies identified during our literature review focus on investigating the impact of carer interventions on parents of young people with AN [56, 57]. These interventions include measures of carer burden and parental distress. However, since these outcomes are the primary targets of the interventions themselves, it does not appear appropriate to include their results within the scope of our research, which aims to assess how FBT, and other therapeutic approaches affect the family unit. Hence, we will only report on studies comparing these types of interventions to TAU, as the intervention alone cannot give relevant information to our research question.

Five controlled studies measuring carer burden and/or distress were included. Carer burden and distress were measured with the EDSIS [58], Experience of Caregiving Inventory [59], Depression Anxiety Stress Scale-21 [60] and the General Health Questionnaire [55] filled by the caregivers. Overall, the studies found burden and distress to decrease or remain stable during treatment, in TAU as well as in participants receiving a carer intervention.

Looking at results in more detail, a controlled study examined the effect of supplementary interventions developed to support parents during existing treatment of families with a YP with AN [61]. As a part of a larger multisite RCT, the study included different forms of TAU depending on collaborative sites. The study found no significant change on parental distress. A pilot randomized study of the same intervention found parental distress to slightly increase from baseline to 12 months FU in TAU [29].

One controlled study compared families receiving TAU to families receiving interventions additional to TAU targeting carer skills [62]. TAU included either multifamily therapy or systemic family therapy. Burden, both measured as general and ED-related distress, decreased significantly in both groups [62]. A sample of the same study investigating the psychometric properties of the German version of the Carer Skills (CASK) showed the same trends [63].

As described earlier (see section on family function) a study compared multifamily therapy with and without participation of YP [50]. The study found negative caregiver experience to significantly decrease in both conditions, and no differences in positive caregiver experiences [50].

#### Attachment

We identified no studies measuring change in attachment after other kinds of treatments for AN in YP.

### Family wellbeing in other treatments: summing up

Eleven studies examined change in family function, family relations including expressed emotion, carer burden and parental stress after therapy where parents were not systematically encouraged to take responsibility for YP´s refeeding. Overall, approximately half of the studies find family wellbeing to improve, while the same proportion of studies find family wellbeing to remain unchanged. No studies find a general deterioration in family wellbeing after treatment without parent-led refeeding. Looking at the different phenomena of family wellbeing separately, family function, family relation/family climate and carer burden/parental distress either improve or remain stable, while EE remains unchanged. The review identified no studies examining change in attachment after other kinds of treatment.

## Discussion

The objective of the scoping review was to provide an overview of the current state of knowledge on whether families in FBT (or similar treatment, where parents are systematically advised to take responsibility for YP’s refeeding) are more or less affected in their family wellbeing over time than families with a young one with AN NOT in treatment with FBT. Of the 30 included studies, however, we identified only three controlled studies directly comparing change in family wellbeing in FBT-like treatment versus non-FBT-like treatment. The three studies examined family function, family communication, and SE but not EE, carer burden, parental stress, and attachment. Therefore, the review question, can only be answered tentatively.

Overall, we do not find indications of a difference in how families in FBT-like treatment and families in other kinds of treatment are affected in their family wellbeing from beginning to EOT. On the contrary, the review finds the same overall pattern in both groups. Thus, based on the research reviewed in this scoping review, *both* FBT-like treatment and treatment where parents are not in charge of refeeding have similar effects on family wellbeing. Apart from one study finding perceived attachment to parents in both YP with AN and in healthy teenagers to decrease over time, no studies find a general deterioration in family wellbeing after any kinds of treatment. How family wellbeing in families with a YP with AN would develop naturally over time, i.e., without treatment, could not be answered, as the review identified no longitudinal studies following YP with AN not in treatment. There are obvious ethical reasons for this lack of naturalistic studies.

It may generate hope for families and clinicians that many studies do find an improvement in family wellbeing over treatment time with families experiencing better communication, fewer family conflicts, less enmeshment, and more adaptability regardless of treatment forms. It is important knowledge for parents and clinicians, that there is no evidence at presence suggesting that FBT, with its active involvement of parents, may harm family wellbeing in general. On the other hand, giving parents an active role does not improve family wellbeing more than other treatments either.

When looking at the change pattern of different aspects of family wellbeing, there is a tendency for some of the measured phenomena to be more prone to improve than others. Thus, family function, family communication, family conflicts and carer burden appear to be susceptible to treatment, while levels of EE in parents may be a more stable trait. This is in line with a view of EE as a moderator of outcome as it is done in most studies of EE. Furthermore, mean level of EE is generally demonstrated to be low in families with a YP with AN [64], why an unchanged level of EE during treatment might be due to a reversed ceiling effect, with treatment not being able to demonstrate a significant reduction in a level, which is low already before treatment.

It is important to note that results reported in the present scoping review express mean findings on the group level. The general tendency may cover a large spectrum of improvement as well as deterioration of family wellbeing for individual families, and clinical practice indicates that some families are greatly affected. The experience of these families must still be heard as they may need different or additional support besides that given in standard FBT. Whether there are family characteristics that might moderate changes in family wellbeing remains an open question and one that could easily be addressed to determine ‘what works for whom’.

Furthermore, before starting treatment YP often have been ill for several months—sometimes years. Hence, it is reasonable to assume that family wellbeing is already affected *before* treatment. Therefore, when many of the studies included in the review find an unchanged level of family wellbeing during treatment, it most likely implies that family wellbeing remains negatively affected at second measure time, compared to how it used to be before AN. This adds to the argument for developing ways to support family wellbeing in the context of AN treatment.

Finally, studies have demonstrated an association between family wellbeing and outcome status [65] and, in extension, clinical experience suggests that when treatment does not work—i.e., when YP continues to be ill, family wellbeing is often increasingly compromised. This effect may be stronger in FBT, because when AN is treated *with* the family, i.e., when the family is the primary instrument for change, treatment failure might be felt as “a failure of the family”. This is a strong argument for supplementary interventions to support carers and family wellbeing, especially when improvement takes longer or seems more difficult than hoped for.

## Strength and limits

The design of the review encompassing a broad range of aspects of family wellbeing, is both a strength and a limitation. Hence, the breadth of the review provides a larger overview of the impact on family wellbeing in FBT vis-a-vis treatment without parent-led refeeding. At the same time, when combining results on very different aspects of family wellbeing, the review runs the danger of comparing or juxtaposing heterogenous phenomena. Furthermore, it was outside the scope of this review to examine how the different operationalizations of family wellbeing may covary or interact.

## What is already known on this subject?

AN in YP is associated with compromised family wellbeing.

## What this study adds?

The review finds that family wellbeing is generally not negatively affected during treatment of YP with AN. Further, impact on family wellbeing is similar in FBT-like treatment and in other kinds of treatments. Thus, family wellbeing either improve of remain unchanged after *both* FBT-like treatments and after treatment where parents are not in charge of refeeding. Since FBT-like treatments are the recommended first line of treatment, it is assuring to parents as well as clinicians, that families in treatments with parent-led refeeding does not appear more affected in their family wellbeing, than families where the parents are not in charge of refeeding. However, in order to ascertain whether treatments with parental-led refeeding might have iatrogenic effects on family wellbeing, our review points to a need for good quality studies directly comparing family wellbeing, including carer burden and parental stress, in FBT with family wellbeing in other treatment types.

### Supplementary Information

Below is the link to the electronic supplementary material.

Supplementary file1 (XLSX 22 KB)

Supplementary file2 (XLSX 26 KB)

## Data Availability

Not applicable.
